# Motor Planning, Not Execution, Separates Motor Memories

**DOI:** 10.1016/j.neuron.2016.10.017

**Published:** 2016-11-23

**Authors:** Hannah R. Sheahan, David W. Franklin, Daniel M. Wolpert

**Affiliations:** 1 Computational and Biological Learning Laboratory, Department of Engineering, Cambridge University, Cambridge, CB2 1PZ, UK; 2 Department of Sport and Health Sciences, Technical University of Munich, 80992 Munich, Germany

## Abstract

Recent theories of limb control emphasize motor cortex as a dynamical system, with planning setting the initial neural state, and execution arising from the self-limiting evolution of the intrinsic neural dynamics. Therefore, movements that share an initial trajectory but then diverge might have different neural states during the execution of the identical initial trajectories. We hypothesized that motor adaptation maps neural states to changes in motor command. This predicts that two opposing perturbations, which interfere when experienced over the same movement, could be learned if each is associated with a different plan even if not executed. We show that planning, but not executing, different follow-through movements allow opposing perturbations to be learned simultaneously over the same movement. However, no learning occurs if different follow throughs are executed, but not planned prior to movement initiation. Our results suggest neural, rather than physical states, are the critical factor associated with motor adaptation.

## Highlights

• Recent studies suggest different plans create different neural states in motor cortex
• When moving in one of two randomly selected opposing force fields no learning occurs
• Linking the fields to distinct follow throughs, even if not executed, allows learning
• Planning is fundamental in the representation of motor adaptation

## Teaser

Sheahan et al. show that planning is more important than execution in allowing separate motor memory formation.

## Introduction

Recent studies of neural coding in motor cortex have emphasized its operation as a dynamical system in which planning involves setting the initial neural state and execution involves allowing the transitory dynamics to evolve from this state (Ames et al., 2014, Churchland et al., 2012, Pandarinath et al., 2015). This suggests that two planned movements that share similar initial kinematics but that diverge later may have fundamentally different neural dynamics, even for the shared kinematic component of the movement. Consistent with this, we have recently shown that opposing dynamics can be learned for movements that are kinematically identical if they are part of a more extensive movement that differs later (Howard et al., 2015). That is, when participants made reaching movements through a force field whose sign depended on the direction of a follow-through movement, they could learn to represent both force fields concurrently for the initial identical component of the movement. In contrast, if the follow-through target was shown (and again associated with the field direction) but no follow-through movement was made, no learning was seen (Howard et al., 2015). Since it is known that motor planning affects neural activity (Cisek and Kalaska, 2005, Riehle and Requin, 1993, Tanji and Evarts, 1976, Wise, 1985), it is possible that planning different follow throughs directly engages separate neural populations or engages the same population by setting the initial neural state of the dynamical systems in motor areas that control movement (Churchland et al., 2012). To test this, here we ask whether it is the planning and/or the execution of the follow-through movement that is essential for the ability to represent opposing perturbations concurrently. We isolate the planning and execution components of the follow through and show that simply having different plans allow multiple motor memories to be learned and expressed for the same physical state of the limb. This suggests that the key to representing multiple memories is to have each associated with a different neural, rather than physical, state.

### Results

**Figure 1:**
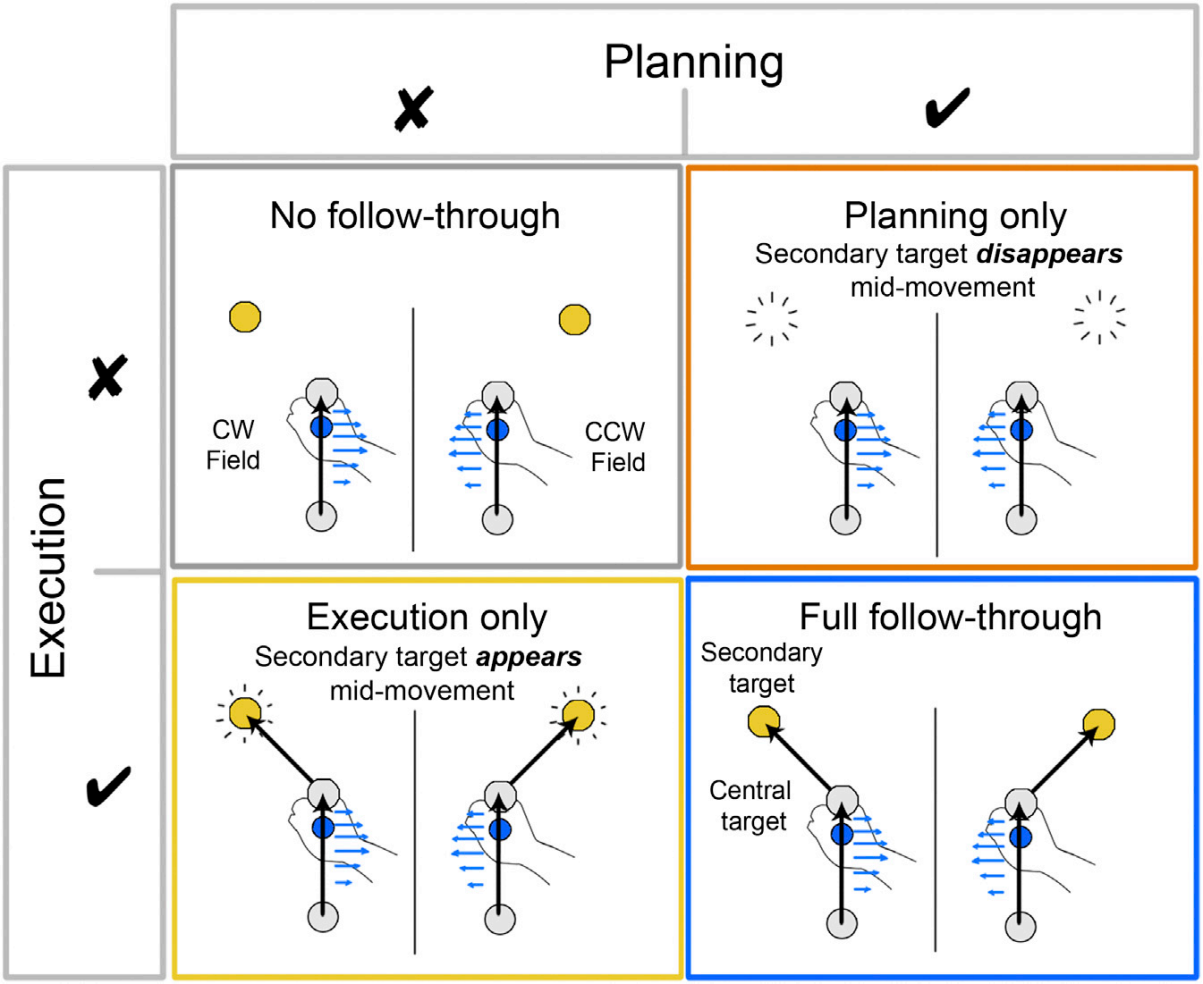
Experimental Paradigm in which Planning and Execution of a Follow-Through Movement Were Factorially Controlled Participants made an initial movement from a starting location (bottom gray circle) to a central target (gray circle). During exposure trials, a velocity-dependent curl force field (force vectors shown as blue arrows for a typical straight line movement to the central target) was applied during this movement, and the field direction, clockwise (CW) or counter-clockwise (CCW), was determined by the secondary target location (at either +45° or −45° to the initial movement direction). A no follow-through group (top left) ended the movement at the central target, whereas the full follow-through group (bottom right) made a follow-through movement, thereby both planning and executing the follow through. For the execution-only group (bottom left), the secondary target only appeared late in the movement to the central target and they were required to follow through. Therefore, this group was prevented from planning the follow through prior to the initiation of their movement. For the planning-only group (top right), the secondary target disappeared late in the movement to the central target and they were required not to follow through. Therefore, this group could plan a follow through before the initiation of the movement, but did not execute it. In all groups, channel trials were used to assess learning and for these trials the secondary target was displayed from the start of the trial. The schematic only shows one of the four possible starting locations used in the experiment.

**Figure 2:**
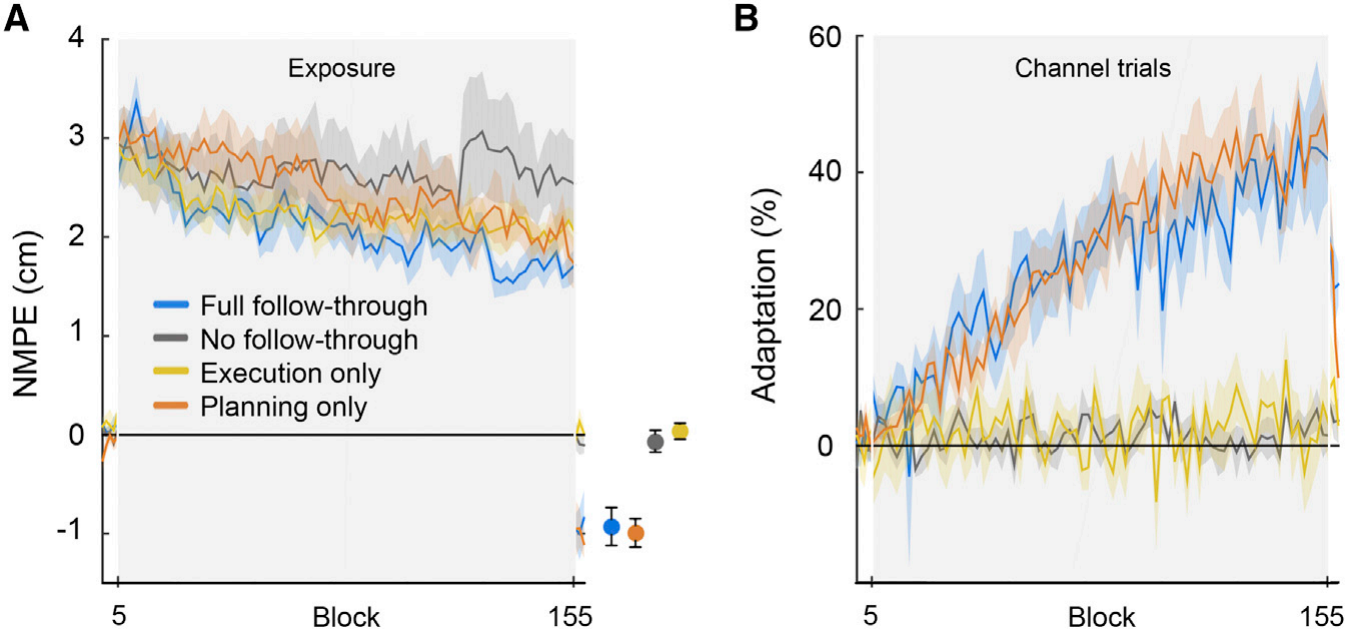
Planning Follow-Through Movements Reduces Interference between Opposing Fields (A) The kinematic error and (B) force adaptation for the full follow-through (blue), no follow-through (gray), execution-only (yellow), and planning-only (orange) groups. Data show mean ± SE across participants for pairs of blocks in the exposure phase (gray region) and for single blocks in the pre- and post-exposure phases. In (A), we show the mean (±SE) of the aftereffects to the right of the panel (separated for clarity).

**Figure 3:**
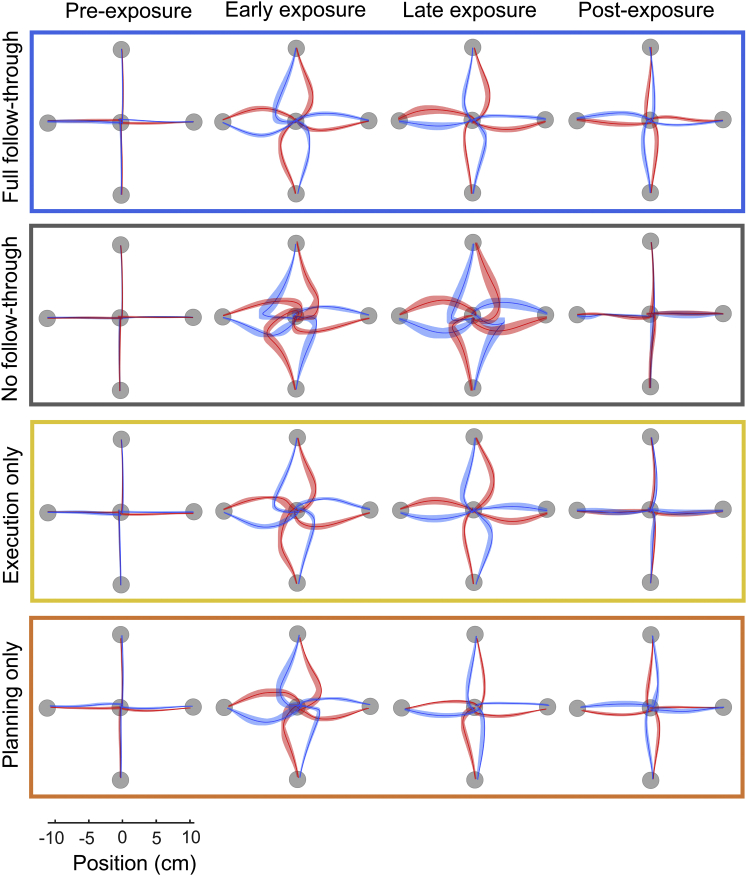
Kinematics across the Groups for Different Phases of the Experiment Hand paths are shown to the central target from the four different starting positions. Paths shown as mean ± SE across participants, for last two blocks of pre-exposure (first column), the first two blocks (second column) and last (third column) two blocks of exposure, and the first two blocks of post-exposure (fourth column). The colors indicate the field direction (blue for CW and red for CCW).

Participants grasped the handle of a robotic interface and made reaching movements from one of four starting locations through a perturbing force field to a central target (see Experimental Procedures). The field direction (clockwise or counter-clockwise) was randomly selected on each trial. For a first group of subjects (Figure 1, full follow through), we associated the direction of the force field with the location of a secondary target (at ±45°). After the target appeared, there was a delay period of 300 ms before a tone cued the participant to initiate their movement. These participants were required to make a second unperturbed, follow-through movement to this target immediately after arriving at the central target. We interspersed the movements in the force field with channel trials, in which the movement was confined to a simulated mechanical channel from the start to central target. This allows us to measure predictive force compensation independently from factors such as co-contraction. As expected (Howard et al., 2015), over the course of 150 blocks (1,200 force field trials) participants reduced their kinematic error (Figure 2A, blue, F_1,5_ = 26.4, p < 0.01; hand paths shown in Figure 3) and showed substantial force compensation on channel trials (Figure 2B, blue, F_1,5_ = 21.5, p < 0.01), reaching approximately 40% of full compensation. In contrast, participants who had the same visual display of the secondary target (which again determined the force field direction) but who did not follow through (Figure 1, no follow through) showed no learning (Figures 2A and 2B, gray; NMPE, F_1,5_ = 0.36, p = 0.574; force adaptation F_1,5_ = 0.08, p = 0.788). This is in accord with many studies that have shown that static cues are insufficient to reduce interference seen when exposed to opposing force fields (Gandolfo et al., 1996, Howard et al., 2012, Howard et al., 2013, Howard et al., 2015). To examine which features of the follow through allowed the separation of motor memories for opposing force fields, in two new groups of participants we isolated execution and planning.

In an execution-only group (Figure 1), the secondary target was not displayed at the start of the trial. Participants initiated the movement in one of the randomly chosen force fields. However, part way through the movement to the central target the secondary target associated with the presented force field appeared (on average 406 ± 14 ms after the cue to move and 270 ± 16 ms after movement initiation), and participants were required to make a follow-through movement to this target. Therefore, subjects executed the follow through but could not plan it prior to the initiation of the movement through the force field. This potentially allowed the participants to retroactively associate the secondary target with the force field. Critically, on the channel trials (presented throughout the experiment) the secondary target was displayed from the start of the trial, allowing us to assess whether any latent learning had taken place based on execution of the follow through. We found that although this group showed a reduction in kinematic error (Figure 2A, yellow; F_1,5_ = 8.77, p < 0.05), this was accounted for by co-contraction as they showed no significant increase in force adaptation (Figure 2B, yellow; F_1,5_ = 0.25, p = 0.638) and no aftereffects in the post-exposure period when the force field was turned off (Figure 2A and Figure 3; t(5) = 0.596, p = 0.577). This suggests that simply executing a follow-through movement to a target, which is uniquely associated with the force field direction, does not allow separation of motor memories.

**Figure 4:**
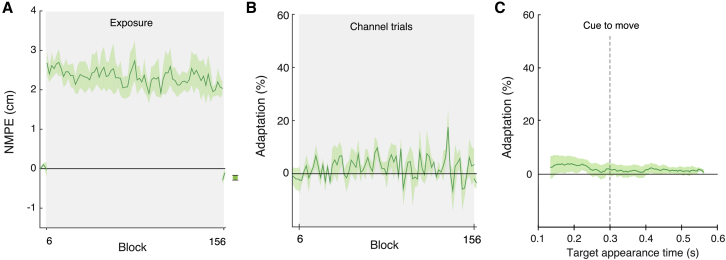
Varying the Appearance Time of the Secondary Target Does Not Facilitate Adaptation in an Execution-Only Group (A) The kinematic error (normalized within this group) and (B) force adaptation (combining 0° and 180° early appearing channel trials). Data show mean ± SE across participants for pairs of blocks. In (A), we show the mean (±SE) of the aftereffects to the right of the panel. In (C), we show adaptation as a function of target appearance time for the second half of the blocks of exposure. Data show mean (±SE) of separate running averages performed for each subject, each with a 150 ms smoothing window at 100 appearance times equally spaced from 0.1 to 0.6 s.

For the execution-only group to have shown learning, they would need to generalize from exposure trials with a target appearing late in the movement to the trials in which the target was displayed from the beginning of the trial (300 ms before the cue to move). To confirm that the lack of learning was not due to an inability to generalize from late-appearing to early-appearing targets, we ran a control in which we included additional channel trials throughout the experiment (see Supplemental Experimental Procedures). On these trials the target appeared at a time uniformly sampled from 300 ms before to 400 ms after the tone, thereby allowing participants to experience a range of target appearance times that should encourage generalization. This group showed no reduction in kinematic error (Figure 4A; F_1,3_ = 4.95, p = 0.113) and no significant increase in force adaptation (Figure 4B; F_1,3_ = 0.758, p = 0.448). In addition, there was no obvious adaptation as a function of target appearance time (Figure 4C). These results suggest that the lack of adaptation in the execution-only group did not result from an inability to transfer adaptation from late target-appearing exposure trials, to early target-appearing channel trials.

To isolate the planning component of the follow through, separate from execution, a fourth group of participants (Figure 1, planning only) made reaches in which the secondary target was displayed from the start of the movement (and as in the other groups a 300 ms delay period was imposed). However, on all exposure trials, when the subjects had reached part way toward the central target, the secondary target was extinguished (on average 301 ± 20 ms after the cue to move and 149 ± 6 ms after movement initiation) and participants were required to terminate their movement at the central target and not follow through. To encourage them to plan the follow-through movement, on channel trials the secondary target remained illuminated and they were required to follow through. We increased the number of channel trials for this group so that one-third of trials were follow through (while maintaining the same number of exposure trials as in the other groups). Even though these participants never executed a follow through on exposure trials, they showed substantial kinematic learning (Figure 2A, orange line; F_1,5_ = 35.4, p < 0.01), a strong aftereffect (Figure 2A and Figure 3; t(5) = −5.05, p < 0.005), and a concomitant increase in force compensation (Figure 2B; orange line; F_1,5_ = 152.1, p < 0.001) to around 40%.

We contrasted the adaptation in the two groups who showed significant learning (full follow through and planning only) using a repeated-measured ANOVA with epoch (two levels: first and last eight blocks in the exposure phase) and group (follow through and planning only). As expected there was a main effect of epoch (F_1,10_ = 85.3, p < 0.0001), but there was no main effect of group (F_1,10_ = 0.02, p = 0.901) or an interaction (F_1,10_ = 0.47, p = 0.508). Therefore, simply planning to follow through leads to learning that is not significantly different from the learning that occurs when both planning and executing a follow through.

To encourage uniformity of movement kinematics, we placed constraints on several features of a trial. A trial was only deemed successful if the hand left the starting circle after the tone sounded and within 1 s, took less than 1.5 s to reach the final target, and remained in the central target for at least 50 ms (success rate was 90.1% ± 1.1% and unsuccessful trials were not analyzed but were repeated). If unperturbed movements to the central target are substantially different for the two possible secondary targets, this could facilitate learning (Howard et al., 2015, Hwang et al., 2003). We examined the kinematics of pre-exposure movements within each group for each secondary target direction (±45°), as well as across groups. For each group and kinematic measure (see Experimental Procedures), we performed a repeated-measures ANOVA on the pre-exposure null trial movements as a function of follow-through direction (±45°). Of the 18 tests, we found only one statistically significant difference (at a conservative p = 0.05 level). That is, for the no follow-through group, the displayed location of the follow-through target (left or right, which they did not move to) led to a small difference in path length to the central target (Δpath length 1.9 mm, p = 0.008). However, such kinematic differences are likely to enhance any learning and given the lack of learning in this group, such a small path length difference does not affect our conclusions.

We also performed comparisons across groups (Table S1). There was no significant difference between dwell time (full follow-through and execution-only groups), lateral deviation, or path length. However, duration (F_3,20_ = 3.2, p = 0.044) and peak speed (F_3,20_ = 5.0, p < 0.01) were significantly different across groups. Post hoc tests revealed that this difference was primarily due to the no follow-through group making faster movements than the other groups (pairwise comparison with three other groups all p < 0.01). However, all measurements of learning take movement speed into account and given that this group is a replication of previous studies (Howard et al., 2013, Howard et al., 2015), such speed differences are highly unlikely to account for a lack of learning. In addition, the planning-only group was faster than the full follow-through group (mean speed difference of 8 cm/s; p < 0.001).

These results show that, when a follow-through movement that is predictive of the field direction is planned, even if not executed, there is substantial reduction in interference.

## Discussion

Our results show that planning different follow throughs, without subsequent execution, allows the learning of two motor skills that normally interfere. Indeed, the amount of learning was not significantly different to when the follow throughs were both planned and executed. Moreover, executing different follow throughs, without being able to plan them from the start of the movement, led to full interference. This suggests that the key to representing multiple memories is to have each associated with a different motor plan.

Our results can be interpreted within the dynamical systems perspective for motor cortex, which places an emphasis on motor planning (Churchland et al., 2006b, Churchland et al., 2012) and suggests a more fundamental role for preparatory activity in motor learning. In this framework, motor preparation during an enforced delay period (400–1,000 ms) involves the setting an initial state of neural activity, from which point the movement naturally evolves through intrinsic neural dynamics. If different movements are planned, delay-period firing rates will be in different initial states and set distinct courses for the consequent evolution of neural and physical activity (Churchland et al., 2006b). A recent study recording in motor cortex from patients with Amyotrophic Lateral Sclerosis confirmed similar neural dynamics in humans compared to non-human primates (Pandarinath et al., 2015). Our results show that simply planning, but not executing, two different follow-through movements results in learning. This suggests that distinct neural states that occur in humans during a delay period for movements with different plans lead to different neural states during the execution of the movement. These different neural states can then be linked to different force outputs, thereby compensating for the opposing perturbations affecting the same physical state of the limb.

Under our hypothesis that different neural states are critical to separate motor memories, there are several other manipulations that, by differentially altering the neural state, could also enhance the representation of multiple skills. Given that the preparatory neural state can depend not only on the planned movement itself, but on how long preparation was sustained (Ames et al., 2014, Churchland et al., 2006b), there may be some ability to differentially adapt otherwise-identical movements if some are preceded by a long delay and others are preceded by no delay. Indeed, recent studies have shown that preparation time can significantly affect the way in which motor learning proceeds (Fernandez-Ruiz et al., 2011, Haith et al., 2015). Moreover, neural activity during planning (delay period of an instructed-delay reach task) in motor regions show differential activity as a function of movement extent (Cisek and Kalaska, 2005, Fu et al., 1993, Kalaska and Crammond, 1992, Messier and Kalaska, 2000, Riehle and Requin, 1989), hand path curvature (Hocherman and Wise, 1991), and peak speed (Churchland et al., 2006a). This suggests that multiple motor memories may be separable based on other planned aspects of the movement.

Several studies have shown that it is easy to learn two opposing force fields if each is applied to a reach to different targets, such as two spatially separated targets (Howard et al., 2013, Hwang et al., 2003, Hwang et al., 2006). A recent study showed that participants can still learn opposing force fields for two spatially separate targets even if vision of the hand is rotated in opposite directions, so that hand kinematics are eventually identical for the two targets but appear visually different (Hirashima and Nozaki, 2012). This led the authors to suggest that planning is the important determinant of interference. However, the use of the visuomotor rotations confounds the effects of state estimation and planning and, moreover, does not allow a dissociation of desired state from plans. When a visuomotor discrepancy is introduced, it leads to a state estimate of the hand’s position that is somewhere between its proprioceptive and displaced visual locations. Many studies have already shown that it is simple to learn opposing perturbations if the state of the hand is different for each (Gandolfo et al., 1996, Howard et al., 2013, Hwang et al., 2003, Hwang et al., 2006). Therefore, the study simply shows that you can learn opposing perturbations if each is associated with a different perceived state of the limb. Our study provides two significant advances on such visuomotor paradigms. First, by using a dynamic perturbation alone our study is the first to show that simply having different motor plans, without the confounding effect of dissociating the visual and physical location of the hand, allows opposing perturbations to be learned. Second, studies of visuomotor learning have not separated the concept of a plan from desired state (as noted in Day et al., 2016). Studies such as (Hirashima and Nozaki, 2012) show that subjects can map different desired states (i.e., left and right targets) to different force fields. However, a desired state is not synonymous with a plan. One can have the same set of desired states arising in different plans, as is the case in our experiment. We show that the same desired states (e.g., hand locations to the central target) can be mapped to two different commands (for the two force fields) when they are part of a movement that has a different overall plan, corresponding to distinct follow throughs (even if not performed). Therefore, previous studies have emphasized the necessity to link different desired states, or the physical or estimated states of the body, to different perturbations to reduce interference. Our results support an alternative and more fundamental hypothesis. That is, what appears to be crucial to separate motor memories is that the underlying plan, and hence neural activity during execution, must be different.

Our study fundamentally asks to what state of the body and/or brain is motor adaptation, in a sense, “attached.” That is, some contexts can tag motor memories, making them immune from interference under other contexts. When the context is the same for two opposing perturbations, adaptation under each perturbation will be driven in opposite directions leading to no net adaptation and, hence, interference. However, if the perturbations are experienced under different contexts, then there will be reduced interference and differential adaptation expressed. A fundamental question is what constitutes different contexts. We show that adaptation “attaches” itself not to the physical situation but to some internal state that differs in anticipation of a forthcoming movement. Based on our results, we propose that situations that lead to differential neural responses in the relevant brain areas will act as different contexts. For example, static cues (e.g., color) linked to opposing force fields have very limited ability to reduce interference (Gandolfo et al., 1996, Howard et al., 2013), suggesting that neural activity in relevant motor regions may not be affected by such cues. In contrast other contexts such as different dynamic cues (Cothros et al., 2009, Howard et al., 2012, Howard et al., 2015), concurrent motion of the other arm (Howard et al., 2010, Nozaki et al., 2006, Nozaki and Scott, 2009, Yokoi et al., 2011), lead-ins (Howard et al., 2012, Wainscott et al., 2005), and follow throughs (Howard et al., 2015) often allow substantial learning. We suggest that such situations that act as contexts may simply be ones that lead naturally to different neural states in motor related regions.

In summary, by isolating the planning and execution components of follow-through movements, we show that it is exclusively the planning component, and not execution, that allows multiple motor memories to be learned and expressed. Our results support a dynamical systems perspective for motor cortex, which emphasizes the primacy of planning over execution in the representation of motor adaptation. This suggests that the critical component that allows separation of motor memories is that the underlying neural states need to be different during the action, and one way this can be achieved is simply by having different plans.

## Experimental Procedures

24 subjects (15 female, 24.8 ± 3.3 years, mean ± SD), with no known neurological disorders, provided informed written consent and participated in the experiment. All participants were right handed according to the Edinburgh handedness inventory (Oldfield, 1971) and were naive to the purpose of the experiments. The protocol was approved by the Psychology Research Ethics Committee at the University of Cambridge.

Experiments were performed using a vBOT planar robotic manipulandum, with associated virtual reality system and air table (Howard et al., 2009). The vBOT is a custom-built back-drivable planar robotic manipulandum exhibiting low mass at its handle. Position and force data were sampled at 1 kHz. The position of the vBOT handle was calculated from optical encoders on the motors. Endpoint forces at the handle of the robotic manipulandum are specified by sending commands to the torque motors. Participants grasped the handle of the vBOT with their right hand, with their forearm supported by an air sled (constraining movement to the horizontal plane). Continuous visual feedback of the subject’s hand position was provided using a computer monitor, projected to the participant via a horizontal mirror, such that a hand cursor (0.5 cm radius) overlaid the veridical hand position in the plane of the movement.

### Paradigm

Participants were divided into four groups (six per group). Participants made reaching movements in a horizontal plane from one of four starting locations to a central target, located approximately 30 cm below the eyes and 30 cm in front of the chest. The four starting locations (1.25 cm radius) were positioned 12 cm from the central target and arranged at 0° (closest to the chest), 90°, 180°, and 270°. During the movement, the robot generated no force (null field trials), a velocity-dependent force (exposure trials), or a spring-like force constraining the hand to a straight-line path to the target (channel trials). On exposure trials, the velocity-dependent curl force field was implemented as:

$$F=b\left[\begin{array}{cc} 0 & 1\\ -1 & 0 \end{array}\right]\;\left[\begin{array}{c} \dot{x}\\ \dot{y} \end{array}\right]$$

where $\dot{x}$ and $\dot{y}$ are Cartesian components of the hand velocity and *b* is the field constant (±15 N.s/m) whose sign determined the direction of the force field (positive = clockwise and negative = counter-clockwise).

Channel trials were used to measure subject-generated forces, a proxy for feedforward adaptation (Milner and Franklin, 2005, Scheidt et al., 2000). On a channel trial, the vBOT produced a spring force field (spring constant of 6,000 N/m, damping coefficient perpendicular to the wall of 50 N.s/m) constraining the subject’s movement to a straight line to the central target.

In addition to the start and central targets, on each trial one of two secondary targets could be displayed (depending on the condition) 10 cm from the central target and positioned at either +45° or −45° relative to the line connecting the starting and central targets. On exposure trials, the direction of the force field applied during the movement to the central target was coupled to the position of the secondary target (e.g., +45° = clockwise; −45° = counter-clockwise). The association between secondary target position and curl field direction was fixed within a participant and counterbalanced across participants. At the end of each trial the vBOT passively moved the hand to the next starting location using a cosine velocity profile.

### Group 1: Full Follow Through

At the start of each trial, one of the starting locations appeared and the hand was passively moved to its location. The central target and one of the two possible yellow secondary targets were then displayed (Figure 1, Full follow through). Subjects were required to remain within the start locations for 300 ms, after which they were cued by a tone to start the movement. We chose this delay period (which was used for all groups) so that the target would be displayed for ∼440 ms prior to movement comparable to the shortest delay periods used in neurophysiological studies of neural dynamics (e.g., 400–1,000 ms delays in Churchland et al., 2012). The movement between the starting location and the central target was through a null field, curl field, or channel and after reaching the central target they continued with a movement to the displayed secondary target. This secondary movement was always made in a null field. Subjects had to remain within the central target for at least 50 ms before following through on to the secondary target. For movement durations from the start position to the secondary target between 400 and 800 ms, a “correct speed” message was displayed; otherwise a “too slow” or “too fast” message was displayed. If subjects moved before the audio cue, took longer than 1.5 s to complete the movement, or took longer than 1.0 s to respond to the audio cue, a mistrial was triggered and subjects were required to repeat the trial.

A block consisted of eight field trials and two channel trials, such that a field trial was experienced at each combination of the four starting positions and two possible secondary target positions (corresponding to the two different field directions). All channel trials were performed from the 0° starting position, one for each of the secondary target positions. The order of trials within a block was pseudo-random.

Before the experiment subjects were given 30 trials of familiarization in a null field. Subjects then performed a pre-exposure phase of five blocks (40 null trials), an exposure phase of 150 blocks (1,200 exposure trials), and finally a post-exposure phase of three blocks (24 null trials). Rest breaks (1.5 min) were provided approximately every 200 trials, with a longer rest break available in the middle of the experiment if required.

### Group 2: No Follow Through

This group only differed from the full follow-through group in that after reaching the central target they were required to stop there, ending the trial (Figure 1, No follow through). At the end of each trial, subjects were provided text feedback of “correct speed” if the movement duration was between 150–250 ms. Otherwise a “too fast” or “too slow” message was displayed.

### Group 3: Execution Only

In the execution-only group, we isolated the effect of executing a follow through without planning it prior to the movement to the central target. On null and exposure trials the secondary target was not displayed at the start of a trial and, instead, the secondary target only appeared once the hand had moved 10 cm toward the central target (Figure 1, Execution only). In piloting we found that this allowed enough time for the participants to make a natural follow-through movement to the secondary target. Importantly, on all channel trials the secondary target appeared from the start of the trial.

### Group 4: Planning Only

In the planning-only group we isolated the effect of planning a follow through without executing it. In contrast to the full follow-through group, once the hand had moved 6 cm toward the central target, the secondary target was extinguished on all null and exposure trials (Figure 1, Planning only). Participants were instructed that if the secondary target disappeared, they were not to execute the secondary movement but instead stop at the central target. We chose 6 cm based on a pilot study so as to trade off the length that we displayed the secondary target during the movement to the central target (as planning could take place during this movement) and the ability of participants to terminate the movement and not overshoot the central target by 3 cm.

Critically, on all channel trials the secondary target did not disappear and subjects performed the full follow through. In order to encourage participants to plan the follow-through movement, we required channel trials for all starting positions (otherwise eight out of ten trials would have been terminated and always terminated for some starting locations). Therefore, in this group we kept the total number of exposure trials the same as the other three groups, but doubled the number of channel trials, including them for each reach direction equally. Therefore a block was 12 trials with 4 channel trials. Across pairs of blocks, we included two exposure trials and one channel trial for every combination of starting location and secondary target position.

Text feedback on trial duration was provided only on channel trials in order to match overall kinematics to the full follow-through group.

### Analysis

A full description of the Analysis is found in the Supplemental Experimental Procedures.

On null and exposure trials, we calculated the maximum perpendicular error (MPE) of the hand from the straight line connecting the starting location to the central target. We normalized the MPE by the peak speed on a trial-by-trial basis to produce NMPE (normalized MPE). On channel trials we measured percent adaptation as the slope of the regression of the time course of the force that participants produced into the channel against the ideal force profile that would fully compensate for the field.

## Author Contributions

H.R.S., D.W.F., and D.M.W., Conception and design, Analysis and interpretation of data, Drafting and revising the article; H.R.S., Acquisition of data.

## Supplemental Information

Document S1. Supplemental Experimental Procedures and Table S1

Document S2. Article plus Supplemental Information
