# Supplementary material for: Motor Planning, Not Execution, Separates Motor Memories
Source: Neuron. 2016 Nov 23;92(4):773–9. doi: 10.1016/j.neuron.2016.10.017 (PMC5167294; doi:10.1016/j.neuron.2016.10.017)
Supplement: Document S1. Supplemental Experimental Procedures and Table S1 [file mmc1.pdf]

**Neuron, Volume *92***

## Supplemental Information

# Motor Planning, Not Execution,

# Separates Motor Memories

**Hannah R. Sheahan, David W. Franklin, and Daniel M. Wolpert**

**Supplemental Information**

**Motor planning, not execution, separates motor memories**

Hannah R. Sheahan, David W. Franklin & Daniel M. Wolpert

## Supplemental Experimental Procedures

### Control experiment

To examine whether the lack of learning in the execution-only group was due to an inability to generalize from late-appearing (exposure trials) to early-appearing (channel trials) targets we ran an additional control. In this control we included trials in which the target appeared at a time uniformly sampled from 300 ms before to 400 ms after the tone. We highlight here only differences from the execution-only main experiment.

We recruited an additional group of 4 subjects (2 females, 31 ± 3.4 years, mean ± s.d.). We increased the size of each block from 10 (8 field trials and 2 channel trials) to 14 (8 field trials and 6 channel trials). Two of the channel trials were, as in the original experiment, with the target appearing at the start of the trial and these were now performed for the 0° and 180° targets. On the remaining 4 channel trials the target appeared at a time between the start of the trial and 700 ms later (i.e. 300 ms before to 400 ms after the tone; or at latest 10 cm into the movement). This time period covers the average appearance times in the execution only group as on exposure trials the target appeared on average 406 ms ± 14 ms after the tone. Across the experiment there were 80 of such random appearance trials for each target and secondary target location. We covered the −300 ms to 400 ms range in 80 equally spaced steps and permuted the order of the appearance times across the experiment for each target and secondary target position (640 trials in total). Across pairs of blocks we ensure that there was a channel trial with a random-appearance time for each combination of starting location and secondary target position.

In addition to adaptation measured on the early target trials we also analyzed adaptation as a function of the time of appearance on random appearance channel trials for the second half of the exposure phase.

### Analysis

For all groups, on null and exposure trials, we calculated the maximum perpendicular error (MPE) as the largest deviation of the hand from the straight line connecting the starting location to the central target. The sign of MPE on each trial was set such that a positive MPE indicated a kinematic error in the same direction as the force field (as would be expected in early learning). Unlike most force-field learning experiments we required participants to reach the central target for a successful trial within a tight time window. This was necessary for the follow through groups who had to pass through the central target on the way to the secondary target. Therefore, to balance the experiment we also required it for the other groups.

In order to adjust for differences in peak speed, either between groups (in particular the no-follow through group moved faster than the other groups) or throughout the experiment as subjects fatigued, which would affect the size of the perpendicular error, we normalized the MPE by the peak speed on a trial-by-trial basis to produce NMPE (normalized MPE). For display purposes we multiplied these normalized values by the average peak speed across all groups and trials.

On channel trials we measured percent adaptation as the slope of the regression of the time course of the force that participants produced into the channel against the ideal force profile that would fully compensate for the field. To do this we extracted a 400 ms (or the maximum available) window of data centred on the time of peak velocity and calculated the force generated by the channel. We used the velocity along the channel to predict the force the vBOT would have applied on an exposure trial. We performed regression (with no intercept) on these times series and expressed the slope as a percentage (slope of 1 = 100%). As the planning only group performed half the number of channel trials at the 0° starting location we included in our analysis the channel trials at 180° as well (the inclusion of only 0° channel trials does not affect the statistical conclusions). For statistical analysis, we averaged the MPE for each subject across consecutive sets of 8 exposure trials. We compared differences in the kinematic error and force compensation between two epochs, the first eight blocks and final eight blocks in the exposure phase, using a repeated measures ANOVA with a main factor of epoch. To assess whether there were any after-effects when the force-field was turned off, we examined whether the mean post-exposure NMPE across subjects differed from zero (t-test).

To display hand paths, we extracted position data from when the hand left the starting location until 50 ms after it entered the central target position. Each path was then linearly interpolated (x and y separately) so as to sample 1000 points equally spaced in time. For each subject, we generated a mean path by averaging the sample paths over trials of interest. To generate a path for a group we calculated the average (and s.e.) of the subjects' paths and plot the mean with shading showing ± s.e (Fig. 3).

We then examined whether the pre-exposure null trial kinematics on the movements to the central target depended on which secondary target position was displayed (±45°). The pre-exposure trials provide a fair comparison as we expect substantial differences during field trials (as the field directions are different for the different secondary targets). Five kinematic measures were extracted for each pre-exposure movement to the central target (Table S1). We calculated the duration, path length and peak speed of the movement. We also calculated the signed lateral deviation from the straight line joining the starting and central target when the hand was midway to the central target. In addition, for the full follow-through and execution only groups we calculated the dwell time that the hand spent within the central target. All duration reports in the Table and main text are mean ± s.e. across subjects.

Within each group we compared these measures for the +45° and –45° secondary targets and also compared these measures across the four groups (collapsed across targets). For each group we performed a repeated measures ANOVA for each kinematic measure as a factor of secondary target direction (2 levels). Here, multiple ANOVAs are more appropriate than a single MANOVA, as we wish to reduce the chances of a type II error. Any differences were explored post-hoc using two-sample t-tests with statistical significance considered at a conservative $p < 0.05$.

| **Measure** | Full follow-through | No follow-through | Execution only | Planning only | $F_{3,20}$ | p |
|---|---|---|---|---|---|---|
| Lateral Deviation (cm) | 0.35 ± 0.01 | 0.30 ± 0.01 | 0.33 ± 0.02 | 0.32 ± 0.02 | 1.36 | 0.27 |
| Path Length (cm) | 10.94 ± 0.03 | 11.12 ± 0.08 | 10.91 ± 0.07 | 11.16 ± 0.13 | 2.12 | 0.11 |
| Duration (s) | 0.324 ± 0.010 | 0.260 ± 0.013 | 0.315 ± 0.014 | 0.302 ± 0.004 | 6.91 | < 0.001 |
| Peak Speed (cm/s) | 43.8 ± 1.9 | 62.7 ± 3.4 | 47.0 ± 2.9 | 51.8 ± 0.9 | 10.94 | < 0.001 |
| Dwell Time (s) | 0.098 ± 0.008 | NA | 0.119 ± 0.008 | NA | 3.11 | 0.09 |

**Table S1 related to Figure 3.**

Analysis of the kinematics for the pre-exposure (null field) trials. For each kinematic measure we examined whether the four groups differed in their mean values. We used repeated measure ANOVAs with a single factor of group. The table shows the mean ± s.e. for each group and the F statistics and p values testing for the difference between the groups. The groups differed in peak speed and duration and this arose from the no-follow through group being faster. NA= not applicable.
